# Learning Damage Representations with Sequence-to-Sequence Models

**DOI:** 10.3390/s22020452

**Published:** 2022-01-07

**Authors:** Qun Yang, Dejian Shen

**Affiliations:** 1 Department of Civil and Environmental Engineering, The University of Auckland, Auckland 1023, New Zealand; 2 College of Civil and Transportation Engineering, Hohai University, Nanjing 210098, China

**Keywords:** structural health monitoring, damage detection, deep learning, Seq2Seq model

## Abstract

Natural hazards have caused damages to structures and economic losses worldwide. Post-hazard responses require accurate and fast damage detection and assessment. In many studies, the development of data-driven damage detection within the research community of structural health monitoring has emerged due to the advances in deep learning models. Most data-driven models for damage detection focus on classifying different damage states and hence damage states cannot be effectively quantified. To address such a deficiency in data-driven damage detection, we propose a sequence-to-sequence (Seq2Seq) model to quantify a probability of damage. The model was trained to learn damage representations with only undamaged signals and then quantify the probability of damage by feeding damaged signals into models. We tested the validity of our proposed Seq2Seq model with a signal dataset which was collected from a two-story timber building subjected to shake table tests. Our results show that our Seq2Seq model has a strong capability of distinguishing damage representations and quantifying the probability of damage in terms of highlighting the regions of interest.

## 1. Introduction

Natural hazards including hurricanes and earthquakes have caused damages to structures and incurred great economic costs in many countries. Post-hazard responses are critical to save lives and mitigate economic losses, requiring accurate and efficient damage assessment. The traditional approach to assessing post-hazard damage is on-site investigations by employing expert inspectors to detect damages. Because of the accessibility to specific locations, such as underneath a bridge deck, is often low, on-site investigations have unavoidable disadvantages in terms of emergency response and post-hazard recovery efforts. Additionally, manual visual inspection is subjective and laborious. Real-time inspection using sensor data to address these drawbacks of on-site investigations have led to the use of emerging technologies within the research community of structural health monitoring (SHM) [1].

The core of real-time inspection technology is dependent on the sensor data. Advancements in sensor technologies make rapidly acquiring rich data possible. Deep learning (DL) models have become a new paradigm in data-driven SHM [2,3]. The key advantage of DL models is that features related to damage patterns can be automatically extracted when detecting damage based on sensor data. The driving forces behind the revolutionary progress of DL-based damage detection can be attributed to the following factors: (1) rich sensor data and powerful computational resources allow large-scale training based on DL models; and (2) the superiority of DL algorithms in extracting features enables data-driven models to outperform conventional inspection approaches in terms of accuracy and efficiency.

Vision-based and vibration-based models are two main data-driven models using DL for detecting damage. Vision-based models implement computer vision technologies to detect damage in structures. Kong and Li [4] proposed a vision-based approach for detecting fatigue cracks. Results show that their method can stably recognize the fatigue cracks irrespectively of ambient lighting conditions. Cha et al. [5] proposes a vision-based method using a convolutional neural network (CNN) for identifying concrete cracks. Their CNN models were trained on a dataset of 40,000 images with 256 × 256 pixel resolutions, achieving approximately 98% accuracy. Cha et al. [6] trained a region-based DL model to recognize four-class damage types, including bolt corrosion, steel corrosion, steel delamination, and concrete crack given a dataset including 2366 images. Gao et al. [7] used transfer learning to avoid overfitting when training Visual Geometry Group (VGGNet) on a relatively small dataset of 2000 images. Their damage detection tasks include component recognition, spalling condition determination, damage level estimate, and damage type classification. Additionally, unmanned aerial vehicles with high-resolution cameras have been deployed with well-trained DL models, achieving the goal of broadening the inspection scope and improving the accessibility [8,9]. These advancements in vision-based methods have successfully addressed the weakness of conventional on-site investigations. However, the main limitation of vision-based models concerns the invisible and internal damage of structures not being recognized. Furthermore, vision-based models aim to identifying different damage classes with high accuracy instead of quantifying corresponding damage states.

Vibration-based models provide promising solutions to the quantification of damage using sensor data such as acceleration responses. These models rely on changes in vibration characteristics due to damage and recognize features that are related to damage from vibration data. Although DL models have been extensively employed to detect damage, limitations are summarized as follows: (1) the probability of damage cannot be quantified according to most proposed methods; (2) most proposed methods rely on a large amount of sensor data, requiring a high computational capacity; (3) analyses regarding damage representations to understand its effects on the quantification of damage are small. Therefore, we propose a Seq2Seq model to quantify the probability of damage given signals from a unknown damage state to address challenges in current data-driven models. We trained the model to learn damage representations with only undamaged signals and then quantified the probability of damage by feeding damaged signals into models. The main contribution of this paper are two-fold: (1) our model only requires the undamaged signals with simple signal processing, improving the computational efficiency; (2) our model is the first to perform quantified damage detection on a 2-story timber building under earthquake excitations.

The rest of paper is organized as follows: Section 2 reviews current progresses of data-driven damage detection in SHM; Section 3 describes the architecture of our proposed Seq2Seq model; Section 4 presents the experiments to verify our Seq2Seq model, including the project overview, training details, and results; Section 5 provides a comprehensive analysis to better understand the model in terms of learning curve, damage representation, and the probability of damage; and Section 6 summarizes the highlights of our Seq2Seq model and conclusions from experiments.

## 2. Related Work

In many studies, DL models have become most promising technology in SHM. Various DL models are extensively used to identify or locate damage. Rafiei and Adeli [10] use a deep restricted Boltzman machine (DRBM) to determine the damage states of a building, with DRBM automatically extracting features from acceleration signals and being useful in identifying both global and local damages. Cha and Wang [11] collected acceleration responses from a steel bridge in the laboratory. First, the continuous wavelet transform and fast Fourier transform were performed to transfer the time series data to the frequency domain. They build an AutoEncoder with a CNN architecture as a feature extractor. Then, a one-class support vector machine (OC-SVM) is trained with extracted features to classify levels of damage. Wang and Cha [12] also proposed an end-to-end damage detection workflow. They designed an AutoEncoder to reconstruct acceleration signals and three damage-sensitive features were then computed according to reconstruction losses. Similarly, an OC-SVM was trained with these damage-sensitive features to predict levels of damage, with an accuracy up to 97.4%. Avci et al. [13] used output-only response data to identify the damage, achieving an accuracy of 99.46% when classifying three-class levels of damage. Abdeljaber et al.’s model [14] can predict binary damage states but only obtain the system-level damage information. Li and Sun [15] trained a CNN model to recognize different levels of damage in a bridge, accurately predicting different severity of damage. Ni et al. [16] built an AutoEncoder with a CNN architecture as a feature extractor by feeding time series signals. Their model was validated using the acceleration responses of a long-span bridge under ambient excitations, detecting damage with a high accuracy. Li et al. [17] proposed a probabilistic structural damage detection algorithm based on Sparse Bayesian Learning and model reduction to infer the stiffness degradation from vibration responses. The algorithm is verified with the vibration responses of two beam structures and a long-span cable-stayed bridge and can reliably detect and quantify various damage scenarios. Yang et al. [18] proposed a novel damage recognition network designed as an encoder–decoder–encoder combination for detecting damage in a building. They trained the model using the Fourier spectra of acceleration signals in the undamaged state to recognize the pattern that is related to damage. Then, the Fourier spectra of the acceleration signals from a unknown damage state are fed into the damage recognition network to quantify the level of damage accordingly. Sony et al. [19] used a recurrent neural network to identify and locate damage based on vibration responses of the building. Their proposed model was verified on two benchmark datasets for binary and multi-class damage classification, respectively.

## 3. Methodology

**Figure 1:**
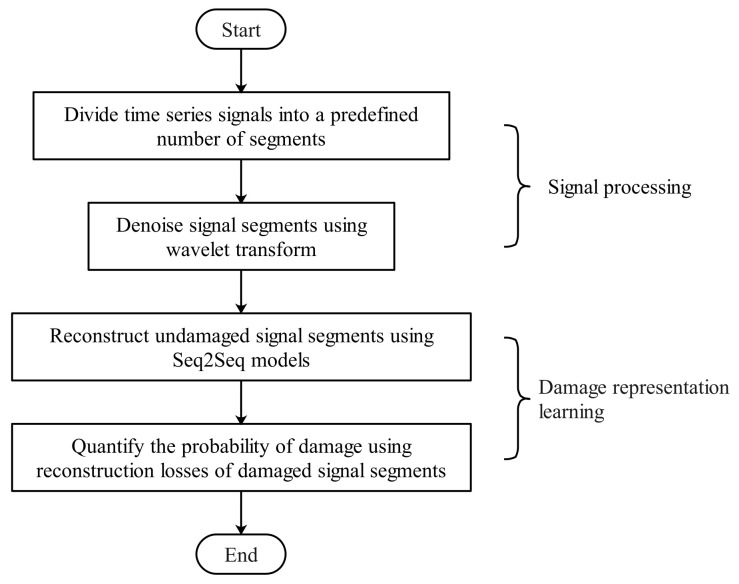
Workflow.

Figure 1 illustrates the workflow of quantifying the probability of damage using Seq2Seq models, including signal processing and damage representation learning. In the first step, the signal processing was required to denoise the original signals with wavelet transform. Then, Seq2Seq models were trained using signals in the undamaged state to learn damage representations and quantify the probability of damage accordingly:

### 3.1. Signal Processing

The signal processing consists of two main steps, namely segmentation and denoising. First, we divided each complete time series signal of *t* seconds that was sampled under a frequency of $f_{s}$ Hz into *n* segments, with the length of each segment being $t\times f_{s}/n$. A proper *n* was determined such that each signal segment included sufficient features for learning representations given a damage state. Then, wavelet transform-based signal denoising was performed to eliminate the effect of noises in signals on the damage representation learning. The process of signal denoising using wavelet transform can be summarized as follows: (1) perform the wavelet transform on each original segments to compute corresponding wavelet coefficients; (2) clean noises by carefully setting a limit to conserve large wavelet coefficients; (3) recover the denoised signals by performing the inverse wavelet transform according to preserved wavelet coefficients from step 2. Denoised signal segments were finally normalized to a range of ±1 for Seq2Seq models.

### 3.2. Damage Representation Learning

**Figure 2:**
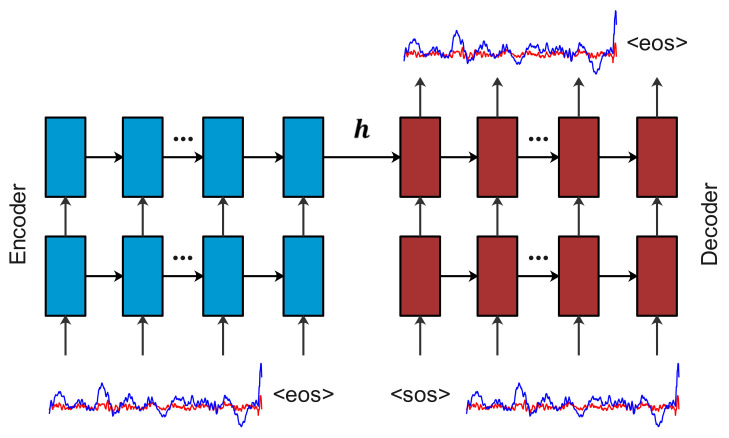
Seq2Seq model—a stacking recurrent architecture for reconstructing segmented signals. Here, <sos> marks the start of a signal; and <eos> marks the end of a signal.

A Seq2Seq model is a neural network that computes a conditional probability of p(y|x) of mapping a source sequence, $x=\{x_{1},\cdots ,x_{n}\}$, to a target sequence, $y=\{y_{1},\cdots ,y_{n}\}$ [20]. The Seq2Seq models were successfully applied for machine translations [20], exhibiting their strong capability of extracting representations of the sequence data. As illustrated in Figure 2, the basic architecture of a Seq2Seq model was comprised of two sub-networks: (a) an encoder that extracts damage representations h for each signal segments; and (b) a decoder that reconstructs a signal value at each time step and hence decomposes a conditional probability as

(1) $$\log p\left(y|x\right)=\sum_{j=1}^{n}\log p\left(y_{j}|y_{<j}\right)$$

A direct option to build a Seq2Seq model is to use a recurrent neural network (RNN) architecture. Alternatively, to prevent the gradient vanishing during the training of long sequences, the long short-term memory (LSTM) unit and gated recurrent unit (GRU) can be used in a Seq2Seq model.

In more detail, one can parameterize the conditional probability of encoding each segmented signal x as

(2) $$p\left(h|x\right)=f\left(x,h_{0};\theta_{p}\right)$$

where *f* determines the probability distribution of the damage representation given the signal, often referred to as the posterior probability, which can either be a vanilla RNN, an LSTM, or a GRU. $h_{0}$ is the hidden state at the initial time step, which is often set as an all-zero vector. $\theta_{p}$ is the parameter set of the encoder.

Then, one can parameterize the condition of decoding each segmented signal with a damage representation h from an encoder as

(3) $$p\left(y_{j}|y_{<j},h\right)=\frac{∥Wh_{j}^{*}{∥}_{2}^{2}}{∥x_{j}{∥}_{2}^{2}}$$

with W being the weight matrix of the fully connected layer that outputs a signal-sized vector. ${∥\cdot ∥}_{2}^{2}$ denotes the 2nd power of the $L_{2}$ norm. Here, $h_{j}^{*}$ is the RNN hidden unit in the decoder, which can be computed as

(4) $$h_{j}^{*}=g\left(x_{j-1},h_{j-1}^{*};\theta_{q}\right)$$

where *g* recursively outputs the current hidden state according to the previous hidden state. The initial hidden state of the decoder $h_{0}^{*}$ is the damage representation h that is extracted from the encoder. Likewise, *g* can be either a vanilla RNN, an LSTM, or a GRU. $\theta_{q}$ is the parameter set of the decoder.

In this work, our training objective is to reconstruct the signal with a Seq2Seq model and hence the loss function is formulated as follows:

(5) $$L\left(x;\theta_{p},\theta_{q}\right)=\sum_{x}-\log p\left(y|x\right)$$

with x and y being the original and reconstructed signals, respectively.

The procedure of learning damage representations with a Seq2Seq model is given in Algorithm 1.
**Algorithm 1** Damage representation learning**Input:** Original signal x**Input:** Number of iterations *N*, signal length *L***Output:** Reconstructed signal y**Output:** Parameter sets of a Seq2Seq model $\theta_{p}$, $\theta_{q}$1:Initialize an initial hidden states $h_{0}$2:Initialize parameter sets of encoder and decoder $\theta_{p}$, $\theta_{q}$3:**for** i≤N **do**4:    Extract hidden states: $h\leftarrow f(x,h_{0};\theta_{p})$5:    **for** j≤L **do**6:        Extract hidden states: $h_{j}^{*}\leftarrow g\left(x_{j-1},h_{j-1}^{*};\theta_{q}\right)$7:        Reconstruct signal: $y_{j}\leftarrow Wh_{j}^{*}$8:    **end for**9:    Update $\theta_{p}$, $\theta_{q}$ by optimizing Eq.(5)10:**end for**11:**return** $\theta_{p}$, $\theta_{q}$

### 3.3. Probability of Damage

We trained a Seq2Seq model with only undamaged signals to learn their representations and hence the model’s reconstruction results are supposed to deviate when inputting damaged signals. The probability of damage according to the extent of deviation is defined as suggested in [18]:

(6) $$P_{d}=1-\exp\left(-e\right)$$

with *e* being the reconstruction loss, which can be computed as follows:

(7) $$e=L(\tilde{x};\theta_{p},\theta_{q})$$

where $\theta_{p}$ and $\theta_{q}$ are the parameter sets of a Seq2Seq model that is trained by undamaged signals, while $\tilde{x}$ is the damaged signal.

## 4. Experiment

In this section, we test the feasibility and effectiveness of using a proposed Seq2Seq model to learn damage representations with a two-story timber building subjected to shake table tests [21]. To distinguish the capacity of our model in terms of learning damage representations, we used a vanilla AutoEncoder, a stacking architecture of multilayer perceptron (MLP), as a baseline model.

### 4.1. Project Overview

The test building has a symmetric plan of 6.10 m by 17.68 m. The first floor height is 3.66 m from the base and the roof height is 3.05 m from the first floor. The total building elevation is 6.71 m [21].

The test building was subjected to real ground motions that represent four increasing hazard levels for the San Francisco site including service-level earthquake (SLE), design-basis earthquake (DBE), maximum considered earthquake (MCE), and 1.2×MCE. To understand the effects of the damage inflicted to the test building as excitation intensity increases, white noise tests were conducted before and after each ground motion test. To quantitatively reflect the severity of damage after each hazard level, we used white noise test WN1 (prior to excitation) to learn damage representations and four white noise tests WN2, WN11, WN17, and WN21 to predict the probability of damage for different hazard levels. The white noise excitation consists of a root mean squared acceleration of 0.03 g. The white noise test data are available in DesignSafe-CI [22].

**Figure 3:**
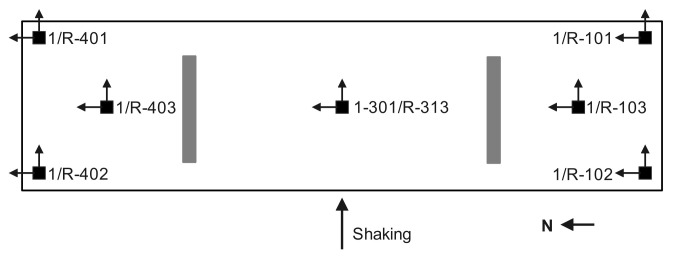
Sensor layout.

Microelectromechanical system (MEMS) accelerometers that are inertial sensors with a low frequency response range but offer lower noise with an acceleration amplitude range of ±5 g were used to measure the dynamic response of the test building [23]. The results reported in [21] show that the amplitude of vibrations induced by the white noise tests is 0.2 g, which does not exceed the measurement range of MEMS accelerometers. The bi-axial accelerometers were placed at seven locations on the first floor and roof, as illustrated in Figure 3.

### 4.2. Training Details

**Figure 4:**
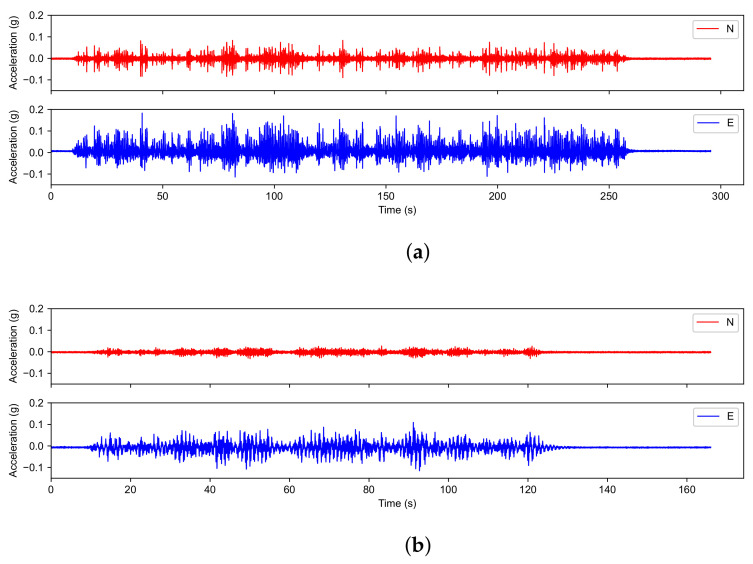
White noise data of sensor 1-101: (**a**) WN1; and (**b**) WN21.

The overall statistical information of datasets is summarized in Table 1. Signals are sampled under a frequency of 240 Hz. The durations of white noise tests WN1, WN2, WN11, WN17, and WN21 are 295.5, 267.0, 176.0, 160.5, and 166.0 s, with the length of signals being 70,902, 64,080, 42,240, 38,520, and 39,840, respectively. White noises are generated by superimposing multi-frequency sinusoidal waves and random noises. The multi-frequency sinusoidal waves are periodical, and the random noises represent the ambient environment excitations. The first white noise test is the longest so that it is beneficial for our model to learn damage representation because more noise can improve model’s robustness and generalization. We divided a signal into 500 segments, as suggested in [10] and pad the last segment with extra zeroes if its length is less than 500 to ensure that each signal segment has the same length. Figure 4 illustrates the white noise data recorded by sensor 1-101 in WN1 and WN21.

Given a segmented signal dataset $D={\left[X_{1},\ldots ,X_{s}\right]}^{T}\in R^{s\times d\times n\times l}$, with *s* being the number of accelerometers (s=14 in this study), *m* being the number of measurement directions of an accelerometers (d=2 in this study), *s* being the number of signal segments (n=500 in this study), and *l* being the length of a signal segment. $X_{i,j,k,:}\in R^{l}$ represents a signal of the $k^{th}$ segment in the $j^{th}$ measurement direction of the $i^{th}$ sensor. For the baseline model, we reshaped D to $D_{b}\in R^{s\times n\times dl}$ by connecting signals in two directions end to end.

Table 2 summarizes the hyper-parameters of training a Seq2Seq model and a baseline model. Our Seq2Seq model has one layer in both encoder and decoder, each with 500 cells, and 2-dimensional embeddings. We used the following settings in training a Seq2Seq model and a baseline model: (a) weights are uniformly initialized in [−1,1]; (b) the hidden size (dimensionality of damage representations) is 128; (c) we trained models using SGD with a momentum coefficient of 0.9; (d) a fixed learning rate of 0.1 was employed; (e) our batch size was 256 and (f) the numbers of epochs were 1000 and 10,000 for a Seq2Seq model and a baseline model, respectively. In addition, dropout with a of probability 0.5 was used for a Seq2Seq model and the normalized gradient was rescaled when its norm exceeded 5.

Our code was implemented in PyTorch and is available at the repository: https://github.com/qryang/Damage-representation (accessed on 1 December 2021). When running on an Amazon Web Service g4dn.xlarge instance, we achieved speed of reconstructing 6000 signals per second. It normally takes 5–6 min to complete training a model.

### 4.3. Reconstruction Results

**Figure 5:**
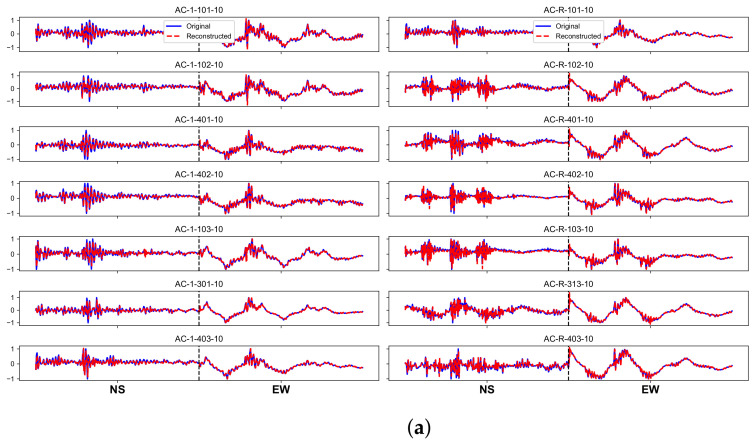

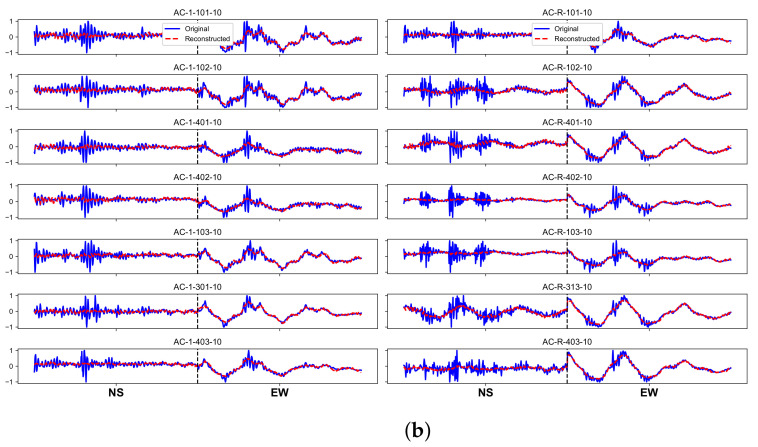
Reconstructed signals: (**a**) Seq2Seq model (LSTM architecture); and (**b**) baseline model.

Figure 5 illustrates the reconstructed results of the 10th segments by using a Seq2Seq model (LSTM architecture) and a baseline model. Our Seq2Seq model has a better result of reconstructing signals compared with the baseline model. The baseline model can only reconstruct low frequency components in the EW direction while losing majority of high frequency components in the NS direction. In contrast, our Seq2Seq model can restore both low frequency and high frequency components in two directions. This can be attributed to memory cells in an LSTM unit effectively establishing the relevance of signals. Better reconstructed results indicate a stronger capacity of learning damage representations.

## 5. Discussion

In this section, we conducted a comprehensive analysis to better understand our Seq2Seq models with respect to learning curves, damage representation, and the probability of damage.

### 5.1. Learning Curve

**Figure 6:**
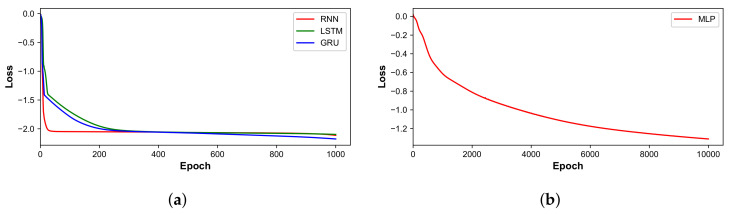
Learning curves: (**a**) Seq2Seq models; and (**b**) baseline model.

Figure 6 illustrates the learning curves of Seq2Seq models and a baseline model, with losses being transferred to a logarithmic scale. For Seq2Seq models, the loss of a vanilla RNN architecture drops quickly at the early stage and smoothly decreases throughout the training process. The LSTM and GRU architectures have a similar convergence speed, converging after 300 epochs. The GRU architecture achieves the lowest loss among all architectures. The baseline model converges after 10,000 epochs but with higher loss compared to Seq2Seq models. The Seq2Seq model converges faster to lower losses than the baseline model but the trade-off is that its time consumption of an epoch is 10 times that of the baseline model.

### 5.2. Damage Representation

The reliability of quantifying the probability of damage by models is heavily dependent on the damage representation. Learning the damage representations of signals is essential to extract useful information when building a decoder to reconstruct signals. In the case of our Seq2Seq model, a probabilistic model, a good damage representation is often one that captures the posterior distribution of the underlying explanatory factors for original signals. Good damage representation is also effectively condensed information extracted by an encoder [24].

We used a 128-dimensional hidden vector extracted by an encoder as the damage representation for both Seq2Seq models and the baseline model. To visualize the distribution of damage representations, we employed the linear discriminant analysis to obtain 2-dimensional damage representations. We chose the GRU architecture as representative of Seq2Seq models as it achieves the lowest loss during the training.

**Figure 7:**
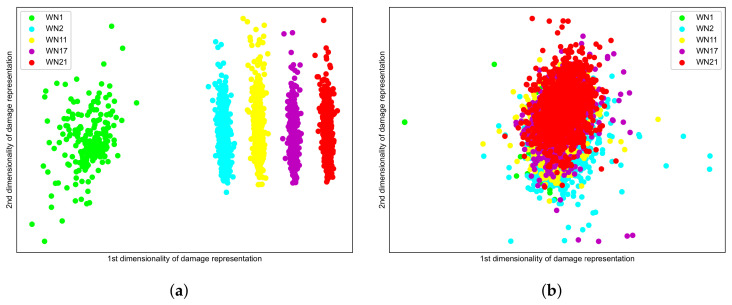
Damage representations: (**a**) Seq2Seq model (GRU architecture); and (**b**) baseline model.

Figure 7 illustrates dimensionally reduced damage representations extracted by a Seq2Seq model with the GRU architecture and a baseline model. The distributions of damage representations in the damaged states (WN2, WN11, WN17, and WN21) are distinct from those in an undamaged state (WN1). However, the distributions of damage representations extracted by Seq2Seq models and the baseline model exhibits different patterns. For a Seq2Seq model with the GRU architecture, damage representations for a heavier damage states clearly scatter with a larger distance to the undamaged state. In contrast, the distribution of damage representations extracted by the baseline model is overlapped without effectively clarifying the different damage states. Damage representations from different damage states contain duplicate information. The better capability of reconstructing signals, especially the high frequency components of our Seq2Seq models, ensures that damage representations contain sufficient information to distinguish different damage states. Therefore, we conclude that a Seq2Seq model behaves better in terms of the discriminability of damage representations than the baseline model.

### 5.3. Probability of Damage

We trained Seq2Seq models to learn damage representations with only undamaged signals and then quantify the probability of damage by feeding damaged signals into models, indicating the severity of damage. Practitioners can reasonably judge the conditions of structures and trace the progress of damage with the assistant of probabilities of damage.

In this section, we also used a Seq2Seq model with the GRU architecture to quantify the probability of damage as comparisons with the baseline model. Table 3 summarizes the results of the probabilities of damage by using our Seq2Seq model and the baseline model. The probabilities of damage computed by our Seq2Seq model show an ascending trend overall, indicating the effectiveness of its capability to quantify damage states. The probabilities of damage computed by our Seq2Seq model and the baseline model are not the exact same. This is due to their different capabilities of learning damage representations.

**Figure 8:**
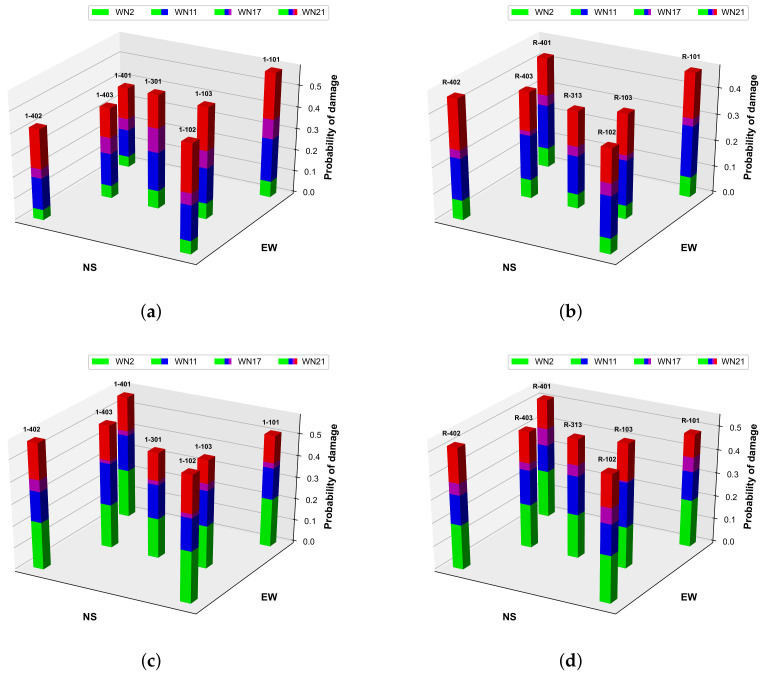
Probability of damage: (**a**) first floor (Seq2Seq model); (**b**) roof (Seq2Seq model); (**c**) first floor (baseline model); and (**d**) roof (baseline model).

Figure 8 illustrates the results of probabilities of damage at places where sensors locate under excitations of increasing intensity by using the Seq2Seq model with the GRU architecture and the baseline model. Probabilities of damage computed by the baseline model are similar irrespectively of regions on the floor. This can be attributed to the representations extracted by the baseline model being almost overlapped, resulting in the model not being able to effectively quantify the distinct probability of damage. Unlike the baseline model, the probabilities of damage computed by a Seq2Seq model can successfully distinguish different regions on the first floor and roof. Probabilities of damage at the center on Level 1 and at the corners on the roof are larger than in other regions. Therefore, our Seq2Seq model has a stronger capability of quantifying the probability of damage in terms of highlighting the regions of interest.

Table 4 summarizes the actual damage levels in terms of the degradation of stiffness, as suggested in [12]. Given that the natural stiffness is proportional to the frequency squared if the test building is considered as a single-degree-of-freedom system for simplicity, we used the results of natural frequencies that are obtained by the modal analysis in [23] to estimate the degradation of stiffness. The stiffness of test building degrades by 25.9%, 32.5%, 44.2%, 46.2%, and 56.8% compared with the undamaged state for four increasing hazard levels. Conceptually, the percentage of degradation of stiffness corresponds to the probability of damage. The core difference is that the percentage of degradation of stiffness is a global index as the representative of damage state, the probability of damage is a local index which can highlight the severity of damage at different regions.

## 6. Conclusions

In this paper, we proposed a Seq2Seq model to quantify the probability of damage given white noise signals. We trained a model with undamaged signals to learn damage representations and then fed damaged signals into a model. Damage representations can recognize different damage states and determine the probability of damage according to the deviation of reconstructed signals.

We verified the effectiveness of using the proposed Seq2Seq model to learn damage representations with a 2-story timber building subjected to shake table tests. To distinguish the capacity of our model in terms of learning damage representations, we used a vanilla AutoEncoder as a baseline model. Results show that our Seq2Seq model can reconstruct signals with a low loss while the baseline model can only reconstruct low frequency components but lose a majority of high frequency components. Compared with the baseline model, our Seq2Seq model has a stronger capability to distinguishing damage representations and quantifying the probability of damage in terms of highlighting the regions of interest.

Our Seq2Seq model is a general model for learning damage representations with the time series data. In future work, one could extend our Seq2Seq model to other areas such as anomaly detection.

## Figures and Tables

**Table 1:** Summary of dataset.

White Noise Test	WN1	WN2	WN11	WN17	WN21
Excitation intensity	NA	SLE	DBE	MCE	1.2×MCE
Duration (s)	295.5	267.0	176.0	160.5	166.0
Length of signal	70,920	64,080	42,240	38,520	39,840
Length of a segment	500	500	500	500	500
Number of segments	142	129	85	78	80
Dataset size	3976	3612	2380	2184	2240

**Table 2:** Hyper-parameter settings.

Model	Seq2Seq	Baseline
Architecture	{RNN, LSTM, GRU}	MLP
Weight initialization	U(−0.01,0.01) ${}^{1}$	
Hidden size	128	
Optimizer	SGD with momentum	
Learning rate	0.1	
Batch size	256	
Number of epoch	1000	10,000

^1^ Uniform distribution.

**Table 3:** Summary of probabilities of damage.

Model Sensors	Seq2Seq ${}^{1}$				Baseline			
	SLS	DBE	MCE	1.2×MCE	SLS	DBE	MCE	1.2×MCE
1-101	0.074	0.276	0.367	0.578	0.222	0.366	0.390	0.513
1-102	0.061	0.226	0.279	0.500	0.238	0.384	0.403	0.571
1-401	0.050	0.179	0.234	0.380	0.220	0.386	0.407	0.562
1-402	0.048	0.196	0.240	0.421	0.217	0.357	0.410	0.574
1-103	0.074	0.239	0.318	0.516	0.197	0.359	0.390	0.495
1-301	0.084	0.263	0.375	0.526	0.183	0.339	0.358	0.483
1-403	0.057	0.211	0.286	0.423	0.200	0.391	0.405	0.562
R-101	0.078	0.275	0.304	0.474	0.203	0.328	0.389	0.485
R-102	0.060	0.219	0.265	0.391	0.204	0.335	0.402	0.538
R-401	0.074	0.243	0.283	0.425	0.202	0.318	0.391	0.514
R-402	0.076	0.236	0.267	0.456	0.193	0.320	0.370	0.518
R-103	0.052	0.225	0.244	0.399	0.180	0.371	0.377	0.531
R-313	0.055	0.202	0.239	0.371	0.189	0.354	0.401	0.510
R-403	0.071	0.243	0.259	0.406	0.188	0.338	0.368	0.501

^1^ GRU architecture.

**Table 4:** Summary of damage levels.

White Noise Test	Natural Frequency [23]	Degardation of Natural Frequency	Degradation of Stiffness
WN1	1.39 Hz	NA	NA
WN2	1.22 Hz	12.2%	25.9%
WN11	1.18 Hz	15.1%	32.5%
WN17	1.11 Hz	20.1%	44.2%
WN19	1.10 Hz	20.9%	46.2%
WN21	1.04 Hz	25.2%	56.8%

## Data Availability

Data are available at https://doi.org/10.17603/ds2-zcb9-ry11 (accessed on 24 May 2019).
